# Progress and Impact of 13 Years of the Global Programme to Eliminate Lymphatic Filariasis on Reducing the Burden of Filarial Disease

**DOI:** 10.1371/journal.pntd.0003319

**Published:** 2014-11-20

**Authors:** K. D. Ramaiah, Eric A. Ottesen

**Affiliations:** 1 Consultant on Lymphatic Filariasis, Tagore Nagar, Pondicherry, India; 2 Neglected Tropical Disease Support Center, The Task Force for Global Health, Decatur, Georgia, United States of America; 3 ENVISION Project, RTI International, Washington, D.C., United States of America; Liverpool School of Tropical Medicine, United Kingdom

## Abstract

**Background:**

A Global Programme to Eliminate Lymphatic Filariasis was launched in 2000, with mass drug administration (MDA) as the core strategy of the programme. After completing 13 years of operations through 2012 and with MDA in place in 55 of 73 endemic countries, the impact of the MDA programme on microfilaraemia, hydrocele and lymphedema is in need of being assessed.

**Methodology/Principal findings:**

During 2000–2012, the MDA programme made remarkable achievements – a total of 6.37 billion treatments were offered and an estimated 4.45 billion treatments were consumed by the population living in endemic areas. Using a model based on empirical observations of the effects of treatment on clinical manifestations, it is estimated that 96.71 million LF cases, including 79.20 million microfilaria carriers, 18.73 million hydrocele cases and a minimum of 5.49 million lymphedema cases have been prevented or cured during this period. Consequently, the global prevalence of LF is calculated to have fallen by 59%, from 3.55% to 1.47%. The fall was highest for microfilaraemia prevalence (68%), followed by 49% in hydrocele prevalence and 25% in lymphedema prevalence. It is estimated that, currently, *i.e.* after 13 years of the MDA programme, there are still an estimated 67.88 million LF cases that include 36.45 million microfilaria carriers, 19.43 million hydrocele cases and 16.68 million lymphedema cases.

**Conclusions/Significance:**

The MDA programme has resulted in significant reduction of the LF burden. Extension of MDA to all at-risk countries and to all regions within those countries where MDA has not yet reached 100% geographic coverage is imperative to further reduce the number of microfilaraemia and chronic disease cases and to reach the global target of interrupting transmission of LF by 2020.

## Author Summary

The mass drug administration (MDA) programme to eliminate lymphatic filariasis (LF) was initiated in 2000. By the end of 2012, the programme was in place in 55 endemic countries. During these first 13 years (2000–2012) of programme implementation, 6.37 billion annual single dose anti-filarial treatments were offered and 4.45 billion doses were consumed by the target populations. This massive programme is estimated to have prevented or cured 96.71 million LF cases that include 79.20 million microfilaria carriers, 18.73 million hydrocele cases and a minimum of 5.49 million lymphedema cases, a 59% reduction of initial LF levels. It is further estimated that, currently, *i.e.* after 13 years of the MDA programme, 67.88 million LF cases remain, including 36.45 million microfilaria carriers, 19.43 million hydrocele cases and 16.68 million lymphedema cases. Progressive reduction in this burden is possible as the programme extends to the endemic countries and regions within endemic countries that have not yet been covered by the MDA programme, and if the morbidity management component of the programme can be effectively implemented.

## Introduction

Lymphatic filariasis (LF) is a disease of the poor that is prevalent in 73 tropical and sub-tropical countries. LF is caused by three species of filarial worms – *Wuchereria bancrofti*, *Brugia malayi* and *B. timori* – and is transmitted by multiple species of mosquitoes. The disease is expressed in a variety of clinical manifestations, the most common being hydrocele and chronic lymphedema/elephantiasis of the legs or arms. People affected by the disease suffer from disability, stigma and associated social and economic consequences. Marginalized people, particularly those living in areas with poor sanitation and housing conditions are more vulnerable and more affected by the disease. Estimates made in 1996 indicated that 119 million people were infected with LF at that time, 43 million of them having the clinical manifestations (principally lymphedema and hydrocele) of chronic LF disease [1].

Earlier severe resource constraints and lack of operationally feasible strategies in the endemic countries left a significant proportion of the LF endemic population living unprotected and exposed to the risk of LF infection. Despite a long-standing and gloomy outlook for these individuals, the situation turned around dramatically in the 1990s for 2 principal reasons: 1) advances made in point-of-care diagnostics and 2) the finding of the long-term effectiveness of anti-filarial drugs given in single doses that permitted development of the strategy of annual two-drug, single-dose mass drug administration (MDA) to control/eliminate LF [2], [3]. As LF had already been postulated to be an eradicable disease [4] and with the success experienced in LF elimination activities in China [5] and elsewhere, the World Health Assembly (WHA) in May 1997 formulated resolution WHA 50.29 urging all endemic countries to increase their efforts and determination to control and eliminate LF. In response, the WHO was able to launch the Global Programme to Eliminate LF (GPELF) in the year 2000, largely because the manufacturers of albendazole (ALB) and ivermectin, two of the principal drugs used in the GPELF MDAs, donated these drugs for as long as needed to eliminate LF [3]. The principal strategy of the programme has been two-fold: 1) to implement MDA programmes in all endemic areas to achieve total interruption of transmission and (2) to provide effective morbidity management in order to alleviate the suffering in people already affected by filarial disease. The GPELF targets elimination of LF, at least as a public health problem, by the year 2020 [6].

The programme to implement MDAs targeting LF (GPELF) completed 13 years of operations in 2012 [7]. With its ambitious goal to eliminate LF by the year 2020, it is essential that progress toward this goal be assessed repeatedly in order to set benchmarks to guide future programmatic planning. *How* to define and assess this progress remains a challenge, but two strategies have been suggested. The first is to measure *reduction in the burden* of LF disease (*i.e.*, hydrocele, lymphedema, microfilaraemia and associated subclinical disease) over the past 13 years – *i.e.*, a clinical perspective; the second is to measure *reduction in the risk of acquiring* infection for populations living in (formerly) endemic areas – *i.e.*, an epidemiologic perspective.

In the present report we have pursued the first alternative – to model the decreased burden of LF (defined for the purposes of our calculations as hydrocele, lymphedema, and microfilaraemia) in order to assess the progress towards LF elimination from inception of the MDA programme through 2012 (*i.e.*, during GPELF's first 13 years). In a parallel study, others have recently modeled the programme's progress from the alternative, risk-of-infection viewpoint (Hooper *et al.*, submitted).

## Methods

A simple ‘force-of-treatment’ model was formulated to estimate the impact of MDA on LF infection and disease.

### Model parameters: Individual countries and regions as the geographic units of assessment

The GPELF aims to provide MDA (using ALB+either ivermectin or diethylcarbamazine [DEC]) to entire endemic populations at yearly intervals for 4–6 years. Such a programme, if implemented effectively (*i.e.* treating at least 65% of the *total population* during each MDA), is expected to interrupt transmission and eliminate LF [8]. Because the status of MDA activities in all of the 73 endemic countries at the time of this analysis (through 2012) ranged from no MDA at all in some countries to full completion of the MDAs in others, for the present study *each country was evaluated separately*. First, programme *impact* was determined for each endemic country; then, the burden of LF *remaining* in each of the five endemic WHO regions – Southeast Asia (SEAR), Africa (AFR), Western Pacific (WPR), Eastern Mediterranean (EMR) and America (AMR) - was calculated by summing the remaining LF burden for all the endemic countries within each region.

### Model parameters: Key elements in assessing programme progress

Calculating progress of the MDA programme under GPELF – whether by *burden* or *risk* estimates – is affected by a number of important specific factors, namely; (1) the number of countries that have successfully completed implementing the MDA programme, (2) the number of countries currently implementing the programme and the geographical coverage or proportion of the endemic population targeted so far in each country, (3) the treatment coverage of the population targeted for MDA in each country, and (4) the duration of the programme (*i.e.*, the number of rounds of MDA implemented) in each country. For the present analysis, all of these data have been sourced from the WHO PC data bank [9].

### Model parameters: Calculation of the decrease in LF burden to assess programme progress

There are 3 essential steps to assessing the decrease of LF burden since 2000: first, the establishment of the LF base-line burden (in 2000); then, estimation of the MDA impact for countries or IUs where MDAs have taken place during 2000–2012; and, finally, estimation of current burden for countries or IUs where *no* MDA has taken place.

#### (i) Establishment of base-line data

The MDA programme under GPELF was started in the year 2000. To quantify the impact of the MDA programme, first, a base-line disease burden was estimated, considering the year 2000 as the base-line year. After extensive review of the literature in the mid-1990s, Michael *et al.* (1996) [1] and Michael and Bundy (1997) [10] estimated the LF prevalence and burden for different endemic regions. LF epidemiology is such that, without specific intervention or environment-altering measures, *prevalence* is unlikely to change over a short period (few years) of time. Hence, for this work the LF *prevalence* during 1996 to 2000 period is considered to remain unchanged. However, the absolute number of people affected by the disease will have increased because of population growth in the endemic areas. Taking the above factors into account, the base-line LF burden was estimated by extrapolating the prevalence data defined earlier [1] to the population of the endemic countries in the year 2000 (Table 1). As the LF burden estimation for individual countries was not always possible due to paucity and availability of data on prevalence, base-line LF burden estimates were made following the earlier approach of Michael *et al.* (1996) [1], and utilizing the convention that all the endemic countries for which no specific information was available, within each endemic region, have an approximately similar average prevalence of microfilaraemia and chronic disease.

**Table 1:** Burden of LF in 1996 and 2000 considered as base-line to understand the impact of MDA (2000–2012) under GPELF.

	LF burden 1996					LF burden 2000				
WHO Region	Total Population endemic countries	Mf carriers	Lymphoedema cases	Hydrocele cases	Total infected	Total Population endemic countries	Mf carriers	Lymphoedema cases	Hydrocele cases	Total infected
SEAR	1335	41.91	9.49	14.53	61.86	1506	47.40	10.74	16.47	70.00
AFR	474	25.78	4.31	9.43	37.06	568	30.91	5.17	11.31	44.44
WPR	1113	11.14	1.52	1.87	13.32	1261	12.62	1.72	2.12	15.10
EMR	100	0.0598	0.0100	0.0199	0.0897	116	0.0700	0.0117	0.0233	0.1050
AMR	179	0.1252	0.0179	0.0179	0.1610	199	0.1397	0.0200	0.0200	0.1796
Total	3200	79.01	15.35	25.87	112.50	3650	91.14	17.66	29.94	129.82

All figures in millions.

The 1996 estimates were based on the work done by Michael *et al.* (1996).

The 1996 data were extrapolated to the populations of each endemic country in 2000 to derive the baseline estimated for GPELF.

#### (ii) Estimation of MDA impact on LF burden for all countries or IUs with MDA in place

Since the decrease in LF burden is a direct result of the treatment provided to populations during the MDA, the model to estimate this burden decrease can be described as a ‘force-of-treatment’ model (see below).

To quantify this force-of-treatment, a ‘treatment index’ (TI) was constructed. The TI is defined as the average number of treatments taken by persons in areas included in MDA. It takes into account three key parameters – the size of the population targeted, the treatment coverage and the number of rounds of MDA implemented. These data can be sourced from the WHO PC data bank [9]. The TI is calculated as *the total number of treatments consumed* divided by the size of the population of IUs included in MDA.

**Figure 1:**
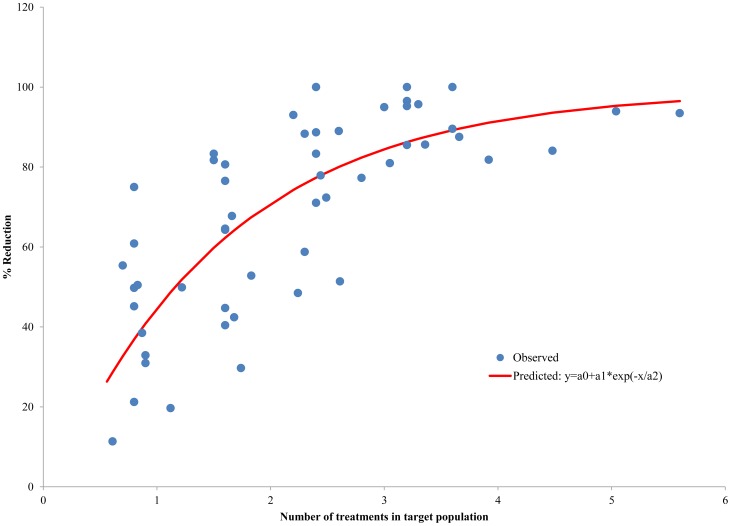
Empiric observations defining the relationship between number of treatments per person and % reduction in Mf prevalence 1 year later.

**Figure 2:**
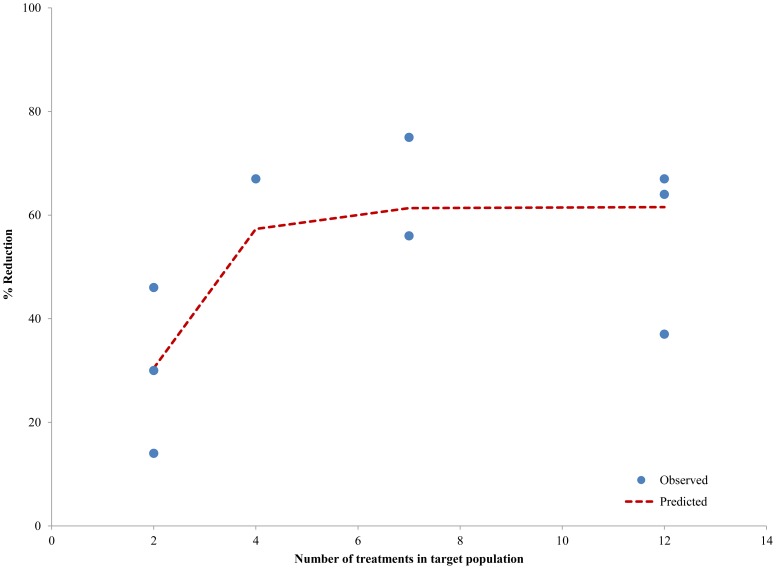
Empiric observations defining the relationship between number of treatments and % reduction in hydrocele prevalence 1 year later.

How to interpret what the TI implies about the effect of the programme's MDAs on LF burden can be determined from considering the *empiric observations reported in earlier studies* of endemic populations treated with the same treatment regimens as those used in the current MDAs; these were reviewed and are summarized below and in Figures 1 and 2.

For microfilaraemia, two of the principal anti-filaria drugs used in MDA campaigns – DEC and ivermectin – have been recognized to exhibit remarkable, rapid effects on decreasing microfilaraemia. The anti-microfilarial effect of both drugs is further fortified when they are administered in combination with ALB, a broad spectrum anti- helminth drug that affects both adult worm viability and production of microfilariae [11]. The impact of treatment on microfilaraemia is evident from the first round of MDA and increases with each round of treatment year after year. While one round of mass treatment has been reported to reduce the Mf prevalence (assessed ∼1 yr post treatment) by 26% to 41%, 5–6 rounds led to 88%–90% reduction [12]–[21]. A review by de Kraker *et al.* (2006) [22] highlighted that both the drug combinations used in GPELF – ALB+DEC and ALB+ivermectin – strongly reduce the LF infection levels, but even 4–6 rounds of single-dose DEC alone can cause reduction of mf prevalence by as much as 86% [13], [23]. Hence, in the present effort to establish the relationship between the number of treatments and the % reduction in microfilaraemia prevalence, results were included from all the community level studies that administered annual single dose treatment (Figure 1), regardless of the specific MDA regimen employed. This empirically derived relationship between the number of treatments given and the decrease in microfilaraemia prevalence (Figure 1), in fact, defines the relationship between the TI and mf prevalence, since the TI is the population-level equivalent of the number of treatments administered at the individual-level. For microfilaraemia, there is a steady increase in reduction of prevalence as the treatment index increases, such that the reduction was close to 95% at a treatment index of about 6.0.

For hydrocele, a similar review was undertaken of available information on the effect that treatment with anti-filarial drugs has on hydrocele prevalence [13], [24]–[29]. Treatment with DEC single dose was common to all of the studies providing results that were used in the analyses. Only one study each evaluated single dose of DEC+ivermectin [13] and ivermectin alone [29] and in both the studies the impact of these drugs was similar to that of DEC. The number of treatments given in these studies ranged from 2 to 12 and in most of the studies treatments were given at yearly or half-yearly interval. A model fitting the non-linear relationship (Fig. 2) was used to define the relationship between the number of treatments and % reduction in prevalence of hydrocele - again, defining the TI for the effect of MDA on hydrocele prevalence (Figure 2). This reduction increased progressively up to 4 treatments, but beyond that the treatment appears to have little additional impact; also, the maximum reduction seen with repeated treatments was approximately 60% (Figure 2).

For lymphedema, different from microfilaraemia and hydrocele, information is scanty on the impact of annual MDA on lymphedema. Studies in Indonesia [30], [31], China [32], and Polynesia [24], all showed reduction in lymphedema prevalence, but all used more prolonged courses or different treatment regimens from those used in the GPELF MDAs. Post-GPELF, three studies evaluated the impact of MDA on lymphedema. In Ghana, one round of MDA with ivermectin and ALB showed no impact on lymphedema [33]. Administration of annual, single-dose DEC for 4 years in Papua New Guinea reduced the lymphedema prevalence by 20% [13]. Seven years of treatment in India showed 14% reduction in lymphedema prevalence in communities treated with annual DEC and 15% reduction in communities treated with ivermectin [29]. In light of these outcomes, a cautious and conservative approach was adopted for estimating the impact of MDA; it is postulated that for a TI of ≥3 (equivalent to nearly 4 rounds of MDA) lymphedema prevalence will be reduced by not more than 14%, the least reduction observed with annual MDA [29]. A TI<3 is considered not to have any effect on lymphedema in adult population groups.

#### (iii) Estimation of burden for the countries or IUs with no MDA in place

For the countries and IUs that do not have MDA in place, the LF burden was extrapolated from pre-MDA, base-line prevalence data. The base-line LF *prevalence* was assumed to remain unchanged, and this prevalence was used to extrapolate the LF burden for the population size of the endemic IUs in 2013.

### Impact of MDA in children

Treatment of LF has been shown to be especially effective and beneficial in children. Prevalence and intensity of childhood infections are relatively low [34], [35], and MDA is particularly effective in clearing them [14], [17], [18], [36]. Assessment carried out after two rounds of MDA suggests that the treatment is able to clear infection in 0–5 year age children [14], [18]; children of 1–10 year age were shown to become free from infection after 2–4 rounds of MDA [18], [20]; and, further, single dose treatment can reverse lymphatic pathology in children [36]. Also, since the MDA exerts an impact on transmission from the first treatment round itself, it offers excellent protection to newborns from acquiring LF [14], [15], [17], [18], [20], [37]. Therefore, for all these reasons the present analysis has considered that the children of 0–5 years in the communities that received one or more MDAs will be free from microfilaraemia and disease. In addition, the children of 0–10 year age in the communities with TI of ≥3 (equivalent to receiving about four rounds of MDA) were considered free from microfilaraemia and disease. Therefore, the impact of the MDAs on LF burden has been treated separately for children and adults.

## Results

### Implementation and progress of the GPELF (2000–2012)

GPELF had a modest start – only 14 of the 81 countries then identified as endemic were able to develop and implement MDA programmes in 2000, the first year of operations, and the target population was 3.2 million. Nevertheless, the programme scaled up progressively, so that by 2005, national programmes were in place in 42 countries with a target population of 610 million [38]. During the subsequent years, further progress has been made. In 2011, 9 countries with a previous history of low prevalence were re-evaluated and declared non-endemic, leaving 73 countries with a combined endemic population of 1,459 million. By 2013, 13 of the 73 endemic countries had completed the MDA phase of the programme and entered into the post-MDA surveillance phase, 42 countries were implementing the programme, but 18 countries still had no programme in place. These 18 countries – 15 of them in the Africa region - account for about 10% of the global endemic population of 1,459 million still living in 73 endemic countries [9].

The status of the programme in terms of number of treatments offered and consumed, as of 2012 in different regions, is summarized in Table 2. Of the 1,459 million endemic population, 975 million individuals (67%) have been targeted by 2012. The 975 million population has been offered a total of 6.37 billion treatments during 2000–2012. The distribution of treatments is noticeably uneven among the two major endemic regions, Africa and South-East Asia. Whereas Africa has 32% of the endemic population, it accounts for only 13% of the total treatments offered, while South-east Asia is home to 62% of the endemic population but accounts for 82% of the treatments offered (Table 3). India alone, with 42% of the endemic population accounts for 71% of the total global treatments offered to date. Of the total 6.37 billion treatments provided, 4.45 billion or 70% of treatments were reported as consumed by the endemic populations.

**Table 2:** Details of treatments given under the MDA programme of GPELF (2000–2012).

WHO Region	Population requiring PCT	Population covered by MDA	Total treatments distributed	Total treatments consumed
SEAR	909	731	5,253	3,651
AFR	468	195	820	588
WPR	44	35	241	161
EMR	28	3	16	15
AMR	14	11	43	34
Total	1,463	975	6,373	4,449

All figures in millions.

Source of data: WHO PCT Data Bank (http://www.who.int/neglected_diseases/preventive_chemotherapy/lf/en/).

**Table 3:** Estimated number of LF cases prevented by the MDA programme under GPELF and current burden in different regions.

WHO Region	Burden in 2000	Projected current burden (2013) assuming no MDA in place	Cases prevented/cured by MDA	Current burden (2013)	% reduction in burden
***W. bancrofti***					
SEAR	63.04	75.23	56.75	18.64	75
AFR	44.44	61.61	18.08	43.42	30
WPR	9.48	11.98	10.48	1.48	88
EMR	0.11	0.14	0.08	0.05	64
AMR	0.18	0.2	0.04	0.15	25
Total	117.24	149.16	85.43	63.73	57
***B. malayi***					
SEAR	6.96	8.21	4.44	3.36	59
WPR	5.62	7.21	6.84	0.79	89
Total	12.58	15.42	11.28	4.15	73
***W. bancrofti*** **+** ***B. malayi*** ** total**	129.82	164.58	96.71	67.88	59

All figures, except % reduction, in millions.

In addition to the 18 countries that had not yet started the programme by 2012, there were also several regionally major endemic countries that had initially launched their programmes but then progressed slowly, principally because of logistic difficulties, funding challenges, lack of political support, civil strife, or, in the case of many Central African countries, the coexistence of loaisis, a contraindication for treating LF with the standard MDA drug regimens [39]. These large countries (including Nigeria, Tanzania, Kenya, Sudan, Papua New Guinea and Indonesia) have an endemic population of 398 million and account for 27% of the global endemic population. (Many of these countries have accelerated their programmes significantly since that time).

### Calculating the impact of the programme

#### (i) Base-line burden

Prior to the commencement of GPELF, in the year 2000, 1.11 billion people living in 81 countries were at risk of LF infection [40], and an estimated 129.82 million people were infected, 90% of them with *Wuchereria bancrofti* and the rest with *Brugia spp*. The 129.82 million infected people could be calculated to have included 91.13 million Mf carriers, 29.94 million with hydrocele and 17.66 million with lymphedema. The South-East Asia Region maintained the highest LF burden and accounted for 54% of the total infections (Table 1).

#### (ii) Calculating the impact of MDA and decrease in burden of LF

The Treatment Index (TI) varied widely across countries, ranging in SEAR from 2.40 in Timor-Leste to 6.40 in Thailand, and in AFR from 0.43 in Cote d Ivoire to 6.03 in Togo. Globally, the highest was 8.36 in French Polynesia, and many of the countries in WPR had a relatively high TI.

Given an unchanging prevalence and steady population growth, had there been no MDA programme during 2000–2012, the estimated number of LF cases in the year 2013 would have been 164.58 million, compared to 129.82 million in the year 2000 (Table 3). The 164.58 million cases would have included 115.65 million with microfilaraemia, 38.16 million with hydrocele and 22.16 million with lymphedema/elephantiasis (Table 4). However, applying the treatment index to the individual country base-line populations results in the estimates that 13 years of the MDA programme (2000–2012), during which 6.37 billion treatments were distributed and 4.45 billion treatments were consumed (Table 2), prevented or cured an estimated 68.22 million microfilaraemia cases, 18.73 million hydrocele cases and 4.32 million lymphedema cases due to *W. bancrofti* infection. The reduction in burden was highest in microfilaraemia cases (67%), followed by hydrocele (49%) and lymphedema (23%) cases. The number of microfilaraemia and lymphedema cases caused by *Brugia spp*. Infections that are estimated to have been prevented was 10.98 million and 1.17 million respectively. Overall, a total of 96.71 million LF cases were prevented or cured (Table 4), equivalent to a 59% reduction (in relation to the estimated number of cases in 2013 if there had been no MDA programme in place).

**Table 4:** Estimated number of different categories of LF cases prevented by the MDA programme under GPELF and current burden.

LF clinical category	LF Burden 2000	Estimated current burden (2013) assuming no MDA in place	Cases prevented/cured by MDA	Current burden (2013)	% reduction in burden
***W. bancrofti***					
Microfilaraemia	80.46	102.46	68.22	34.25	67
Hydrocele	29.94	38.16	18.73	19.43	49
Lymphedema	14.84	18.72	4.32	14.41	23
Total	117.24	149.16	85.43	63.73	57
***B. malayi***					
Microfilaraemia	10.67	13.19	10.98	2.2	83
Lymphedema	2.82	3.44	1.17	2.27	34
Total	12.58	15.42	11.28	4.15	73
***W. bancrofti+B. malayi***					
Microfilaraemia	91.13	115.65	79.2	36.45	68
Hydrocele	29.94	38.16	18.73	19.43	49
Lymphedema	17.66	22.16	5.49	16.68	25
Total	129.82	164.58	96.71	67.88	59

All figures, except % reduction, in millions.

As a result of the MDA programme, the global prevalence of LF can be calculated to have declined from base-line level of 3.55% to 1.47%, equivalent to 59% reduction. The current *global* prevalence of microfilaraemia is 0.79%, of hydrocele is 0.42% and of lymphedema, 0.36%. The overall prevalence of LF in Africa continues to be higher at 5.51%.

It is estimated that this current global LF burden, after 13 years of MDA programme, includes 36.45 million cases of microfilaraemia, 19.43 million cases of hydrocele and 16.68 million cases of lymphedema, totaling an overall estimated burden of 67.88 million cases. Of these cases, 64% are in SSA and 32% in SEAR, compared to 34% and 54% respectively during the baseline period (Table 3).

#### (iii) Other potential benefits

Acute episodes of adenolymphangitis (ADL) are also a considerable health problem among the LF affected communities, as they may cripple affected individuals for up to a month at a time. Their incidence is much higher in those affected by the chronic disease conditions of hydrocele and lymphedema (accounting for 83% of identified episodes) [41], [42]. On average, people with lymphedema suffer from 2.3 episodes and hydrocele patients suffer from 1.4 episodes per year [41]. As 13 years of MDA programme prevented or cured 18.73 million hydrocele cases and 5.49 million lymphedema cases, this is estimated to have also averted 38.85 million ADL episodes per annum.

## Discussion

Prior to the GPELF, efforts to control LF met with little success, largely because of the lack of feasible and affordable strategies. Even most of the countries that initiated control programmes in the 1950s could make only marginal progress because of the relatively low priority for LF control and lack of feasible, scalable control strategies. The advent of preventive chemotherapy-based annual MDA programmes and the launching of GPELF provided great stimulus toward the control and elimination of LF and its very significant health and socio-economic consequences. Single dose treatment was shown to be very effective against LF infection [2], and mass administration of such single dose treatment was shown to be both broadly feasible [43] and comparatively inexpensive [44], [45]. Availability of donated drugs [46] and the implementation support by international organizations and aid agencies [3], [47] provided further impetus to launch the MDA programme. These factors have enabled as many as 55 countries to undertake national MDA programmes targeting LF elimination. In these countries, an unprecedented 6.37 billion treatments were made available during 2000–12 period [9], making the preventive chemotherapy for LF elimination one of the largest ever public health interventions. The scale of the programme also highlights not only the positive response of endemic countries to accept the challenge of implementing interventions that are ‘simple’ and feasible but also the ability of these countries – some of them among the least resourced – to implement these very large-scale public health programmes successfully.

Given all of this *implementation* success, it is now essential that the disease-specific health impact of these programmes be assessed as well. While there are, indeed, many important clinical consequences of LF infection (including renal pathology [48], acute episodic ADL [41], [42], [49] and others [50], because the manifestations most frequently measured are microfilaraemia, hydrocele and lymphedema/elephantiasis, it is these that we have tracked in modeling GPELF's impact on the burden of LF disease.

LF infection in individuals goes through different phases, beginning with pre-patent infection, then progressing to microfilaraemia, acute manifestations and chronic disease. The anti-filarial drug regimens used in the GPELF – ALB+either DEC or ivermectin – exhibit excellent microfilaricidal effect even in single doses at both the individual and community level [12]–[21]. Hence, as expected, thirteen years of an MDA programme that delivered 6.37 billion treatments with an intake of 4.45 billion treatments (Table 2), has prevented or cured an estimated 79.20 million microfilaraemia cases in the endemic countries. Currently, as projected in this study, there are still an estimated 36.45 million Mf cases, a figure that is still high but that would have been an astounding 115.65 million cases, had there not been an MDA programme under GPELF (Table 4). This also means that the consequences of microfilaraemia, which include LF progression to chronic disease in a proportion of those 79.20 million people, were averted as well (see below).

The direct effects of treatment with anti-filarial drugs are less remarkable against chronic disease manifestations than on microfilaraemia. However, several studies have shown that treatment does, indeed, have significant impact on chronic disease manifestations, ranging from reversal of early disease signs and symptoms to actual reversal of some of the chronic lesions. The presence of adult worms alone is sufficient to cause hydrocele [50] and reduction in adult worm burden is understandably able to lead to reduction in hydrocele prevalence. The anti-filarial drugs used in the MDA programme - albendazole plus ivermectin, as well as DEC alone or with ALB - exhibit at least partial adulticidal effect, thereby reducing the adult worm burden [51], [52] and hydrocele prevalence in treated individuals [24]–[28]. When the relationship between treatment doses and the reduction in hydrocele prevalence (Fig. 2) was extrapolated to the MDA programme, a reduction of 18.73 million hydrocele cases was projected (Table 4) - reflecting both the prevention of new hydrocele cases, particularly in the younger population, and the cure of hydrocele in a proportion of those older, already affected individuals.

Relatively fewer studies have examined the impact of single- or repeated, annual single-dose treatment on lymphedema and elephantiasis. In Indonesia and Tahiti very high reduction i.e. 68% to 80% in lymphedema prevalence was observed after 82 mostly weekly doses and 12 monthly doses respectively [24], [30]. However, the impact of typical *annual* MDA was critically evaluated only in two studies, one each in India and Papua New Guinea. The reductions were 14% after 7 rounds of MDA in the Indian study using DEC alone [29], and 20% after 4 rounds of MDA, using DEC alone in Papua New Guinea [13]. Taking various studies into account, we assumed conservatively that in communities with TI of 3 and above, which is equivalent to nearly four rounds of MDA, a 14% reduction in lymphedema prevalence is achieved. This conservative approach was adopted not only to avoid overestimation of the programme impact but also because most of the MDA implementing countries have not yet established robust national morbidity management programmes, whose benefits on disease-improvement will be substantial from controlling the bacterial superinfection of affected limbs that is essential to the progression of elephantiasis [50]. Our analysis suggests that, even despite this conservative modeling approach, an estimated 5.49 million lymphedema cases were prevented or cured by the MDA programme in its first 13 years (Table 4). While those born during and after transmission has been interrupted will have no risk of lymphedema, from a practical standpoint it will still be essential to institute morbidity management programmes in order to achieve significant relief for those already affected.

The estimated disease-specific impact of 13 years of the GPELF (Table 4) has been calculated on the basis only of microfilaremia, hydrocele and lymphedema/elephantiasis, but it is clear that other very significant effects on reducing LF burden have been achieved as well. For example, 79.2 million cases of microfilaremia were projected to have been averted by the Programme (see above), and since nearly 50% of Mf carriers show renal abnormalities which resolve with treatment [48], several million Mf carriers can be recognized to have benefited from resolution of such renal abnormalities as well. Also, since the transmission of LF is generally proportional to the number of Mf carriers and the intensity of microfilaraemia in communities [53], such a significant reduction in the number of Mf carriers also means considerable decrease in transmission of LF in the treated communities; and, of course, transmission reduction and its ultimate interruption determine the elimination of LF, the principal objective of the MDA programmes. Similarly, the projected reduction in chronic LF cases – 18.73 million hydrocele cases and 5.49 million lymphedema cases– is estimated to have averted 39 million acute ADL episodes in endemic areas. This is expected to result in significant relief to the infected population, as ADL, though transient, inflicts severe suffering, makes affected people bed ridden [41], [42], [54]–[56] and requires recuperation from these episodes often extending for weeks at a time.

In an earlier study [57], it was estimated that eight years of MDA, under which >1.9 billion treatments were delivered, prevented 7.4 million cases of hydrocele and 4.3 million cases of lymphedema. While these estimates on the number of hydrocele cases prevented are similar to the estimates in the present study, there is less agreement on the number of lymphedema cases prevented. The estimated 5.49 million lymphedema cases prevented in this study, after 13 years of MDA and delivery of 6.37 billion treatments, was lower, likely because of both the different strategies for calculating the effects and the conservative approach adopted in assessing the impact of MDA on lymphedema. The estimated 5.49 million lymphedema cases prevented in this study was a *minimum number*, and the actual reduction may be much higher.

Of the various factors influencing the outcome of MDA programmes, treatment coverage is particularly important [8]. In this study, the impact of MDA was assessed using the *reported* treatment coverage – *i.e.* the treatment coverage reported by the country level programme managers and compiled in WHO's PC data bank [9]. There are, however, a number of reports suggesting that the programme-reported treatment coverage in the South-east Asia region, particularly in India, may be higher than the actual treatment coverage in the communities. For example, while programme-reported treatment coverage in India was generally in the range of 58% to 90%, various independent studies showed treatment coverage that varied widely and ranged from <20% to >90% in different parts of the country [58]–[74]. The data from these published studies give rise to an average ‘evaluated’ treatment coverage rate of 51.0%, less than the 71.33% average reported national coverage [9]. Since the TI used to calculate programme impact in our model incorporates programme coverage, it is necessary to understand the effect of this difference between reported and evaluated coverage. For India, the TI based on reported coverage was 5.27, but only 4.21 when based on ‘evaluated’ coverage – a difference of 20%. Interestingly, however, when those different TI's were applied to the model (Figs. 1 & 2), the effect was minimal, because for TI's >4, little or no additional benefit was achieved on the 3 parameters measured (microfilaraemia, hydrocele, lymphedema/elephantiasis). In other words, the initial rounds of MDA will exert greater impact on these manifestations compared to later rounds, a finding already reported empirically and shown in various studies [12], [13], [15], [17]–[20]. However, if the treatment coverage rate *is* high, a higher TI can be achieved in the early rounds of the programme, and fewer rounds of MDA may be required to maximize both impact and cost-effectiveness.

It is possible that preventive chemotherapy as well as other interventions implemented against other vector-borne diseases have added to the impact of LF MDA and caused further reduction in LF burden in some countries. Principal among these other interventions are the ivemectin distribution under the African Programme for Onchocrciasis Control (APOC) and the malaria control measures of insecticide treated nets (ITN) and indoor residual spraying (IRS). Currently, ivermectin is distributedfor onchocerciasis control in as many as 26 countries in Africa, covering nearly 130 million population [75]. Most of the 26 countries are co-endemic for LF also and while less than half of this LF-endemic population is under specific treatment as part of the GPELF, many are likely receiving benefit from the ivermectin being used for onchocerciasis control, as has been demonstrated specifically in a number of countries in West Africa [76]–[80]. Similarly, the malaria control measures have been shown to reduce LF transmission considerably and remain promising adjuncts to the MDA of the GPELF activities [81]–[83].

While these coincident intervention measures have, and will continue to have, positive impact on the LF elimination efforts, quantification of their impact remains a daunting challenge. The reduction in LF burden achieved during the GPELF's first 13 years is almost certainly higher than shown through our analyses both because of the additional, on-going intervention measures and because of our conservative approach to estimating the impact on chronic disease.

Though, there can be little question that impressive gains in decreasing LF burden have been achieved as a result of 13 years of MDA in the GPELF, still, however, a considerable burden of LF remains – estimated at 36.45 million Mf cases, 16.68 million cases of lymphedema and 19.43 million cases of hydrocele (Table 4). Extension of MDA to all at-risk countries and to all regions within those countries where MDA has not yet started is absolutely necessary to reduce the number of microfilaraemia cases and transmission. Such an extension of MDA will also reduce a *proportion* of hydrocele and lymphedema cases, but the burden of LF disease needs also to be approached directly. Techniques for effective morbidity management – both medical and surgical – are available but not nearly so widely implemented as they could or should be. The present model's calculations take into consideration only those burden-reducing benefits coming *pari passu* with MDA implementation. When appropriate morbidity management strategies are finally introduced and accelerated, the burden of LF disease will fall even more dramatically (and the model can be adapted accordingly).
